# Urinary fatty acid binding protein 2 as a biomarker of intestinal injury in pediatric celiac disease

**DOI:** 10.3389/fped.2026.1838294

**Published:** 2026-06-10

**Authors:** Vijay Mehta, Nidhi Kapoor, Chloe Milliron, Noah Stoeckel, Edwin Ballelos, Katrina Tiqui, Chirajyoti Deb, Bassam Abomoelak, Mary Schreck, Devendra Mehta

**Affiliations:** 1 Pediatric Gastroenterology, Orlando Health Arnold Palmer Hospital for Children, Orlando, FL, United States; 2 Specialty Diagnostic Clinical and Translational Research Lab, Orlando Health Arnold Palmer Hospital for Children, Orlando, FL, United States; 3 University of Central Florida, Orlando, FL, United States; 4 Orlando Health Medical Group Pediatric Specialty Practice Resources, Orlando, FL, United States

**Keywords:** autoimmune enteropathy, biomarker, intestinal integrity, intestinal permeability test, leaky gut, mucosal damage

## Abstract

**Objectives:**

Celiac disease (CeD) is an autoimmune enteropathy triggered by gluten in genetically predisposed individuals, causing intestinal damage and impaired gut barrier integrity. Current diagnostic methods, such as serology and intestinal biopsy, are costly and invasive. Intestinal fatty acid binding protein (FABP2), exclusively expressed in the cytosol of mature enterocytes and released during injury, offers a potential non-invasive alternative biomarker. This study aimed to evaluate urinary FABP2 levels in pediatric patients undergoing gastrointestinal assessment or CeD follow-up to determine its potential as a non-invasive biomarker of intestinal integrity.

**Methods:**

In this retrospective cross-sectional study, urinary FABP2 was measured in 103 pediatric patients evaluated for intestinal permeability using the lactulose/mannitol test. Based on serology and symptomatology, patients were categorized as controls (*n* = 65), CeD on gluten-free diet (GFD) > 6 months (*n* = 26), or active CeD (*n* = 12). Associations between FABP2 levels, serology, symptoms, and diagnosis were analyzed.

**Results:**

FABP2 levels were significantly elevated in active CeD patients (47.7 pg/mL) compared with controls (9.65 pg/mL) (*p* < 0.05), but not in CeD patients on GFD (15.0 pg/mL). A receiver operating characteristic curve analysis confirmed the diagnostic potential of FABP2 as a biomarker for celiac disease (area under the curve = 0.7269; cutoff = 23.7 pg/mL; sensitivity = 66.7%, specificity = 76.9%).

**Conclusion:**

Our findings indicate that urinary FABP2 reflects intestinal epithelial injury and may serve as a non-invasive biomarker for assessing gut integrity and disease activity in CeD. However, larger prospective studies with histological correlation are needed to validate its diagnostic utility.

## Introduction

Celiac disease (CeD) is an immune-mediated enteropathy triggered by the ingestion of gluten in genetically susceptible individuals, with a prevalence of ∼1% of the global population (1, 2). Key, well-defined contributors to CeD pathogenesis include genetic factors HLA DQ 2 and HLA DQ8, an environmental trigger (gluten prolamines), and the involvement of autoantigens, in particular tissue transglutaminase 2 IgA (tTG-IgA 2) (3).

The intestinal epithelium comprises several different cell types, such as enterocytes, paneth cells, and goblet cells. These cells, along with intracellular tight junctions, adherens junctions, and desmosomes, form a tight barrier toward the intestinal luminal milieu (4). However, as CeD progresses, it could lead to malabsorption, inflammation, intestinal cell damage, and compromised gut barrier integrity (5). Previous studies have shown ongoing inflammation and heightened intestinal permeability in patients with conditions such as celiac disease, type 1 diabetes mellitus (DM), and bacterial overgrowth (6, 7). In CeD disease, monitoring gut integrity is important to detect persistent mucosal damage, assess treatment adherence, and prevent complications such as malabsorption and disease progression, including T-cell lymphoma.

There are several diagnostic methods to determine gut integrity, such as histological analysis of intestinal biopsies, fecal calprotectin, and oligosaccharides absorption tests. However, these methods are complex, time-consuming, and often difficult to use in routine clinical practice.

Currently, CeD is diagnosed through a combination of serologic tests [such as tTG-IgA and deamidated gliadin peptide (DGP)-IgA], HLA testing performed in selected cases, and confirmatory small-bowel biopsy (8). Once diagnosed, CeD patients are managed through lifelong adherence to a gluten-free diet (GFD). However, despite the known benefits of a GFD, including symptom resolution, normalization of serologic markers, and mucosal healing, monitoring disease activity and dietary compliance remains a clinical challenge (9). This is largely due to the lack of reliable, non-invasive biomarkers that accurately reflect real-time intestinal injury and healing. Furthermore, while serologic markers offer high sensitivity and specificity for CeD diagnosis, these antibodies persist for extended periods due to their long half-lives, which are estimated to be 6–12 months, rendering them unreliable for real-time monitoring of mucosal recovery or dietary adherence (10, 11). Thus, there remains a critical gap in the clinical toolkit for diagnosing the condition, tracking disease activity, and guiding individualized patient care.

Intestinal fatty acid binding protein (IFABP or FABP2) is a 15-kDa cytosolic protein specific to enterocytes, released rapidly into circulation upon intestinal epithelial injury (12, 13). Studies have demonstrated elevated serum FABP2 levels in patients with active CeD compared with healthy controls and a strong correlation with histologic injury as measured by the Marsh classification (14–16). In addition, serum FABP2 levels have been shown to decline rapidly in pediatric CeD patients following adherence to a GFD, supporting its potential as a dynamic biomarker (17, 18). A recent study by Logan et al. further validated the superiority of FABP2 over tTG-IgA in monitoring GFD adherence (19).

While prior studies have focused primarily on serum FABP2, emerging evidence suggests that FABP2 can also be reliably measured in urine, offering a non-invasive alternative for monitoring intestinal damage (14, 20, 21). However, few studies have evaluated the utility of urinary FABP2 as a non-invasive biomarker for diagnosis, GFD adherence, and mucosal healing in pediatric CeD populations.

Thus, in this study, we aimed to compare urine FABP2 levels among children with active celiac disease (AC), those with celiac disease on a gluten-free diet (CeDGFD), and controls.

## Methods

### Ethics statement

This single-center retrospective study was approved by the Orlando Health Institutional Review Board (IRB) (IRB #24.034.02) on 29 May 2024 under expedited review with a waiver of informed consent. This study utilized remnant clinical specimens retained in the laboratory following standard-of-care lactulose–mannitol testing performed between January 2020 and April 2024.

### Patients

This retrospective study was conducted at Arnold Palmer Hospital, Orlando, Florida. Pediatric patients who underwent clinically indicated lactulose–mannitol intestinal permeability (leaky gut) testing between January 2020 and April 2024 were included in this study. Urine samples were collected and sent to our specialty diagnostic and translational research lab at Orlando Health for routine clinical lactulose–mannitol intestinal permeability testing. Remnant fluid was frozen at −80 °C until further analysis for FABP2. Freeze–thaw cycles were minimal, and all samples were treated equally. Clinical data of patients were obtained via EMR when able. As electronic medical record (EMR) had been transitioned in 2021, patients who were diagnosed elsewhere or prior to the EMR switch may have had incomplete lab values, including tTG-IgA.

Patients were grouped into AC, CeDGFD, and controls, based on a combination of serology (positive antibodies for tTg-IgA), endoscopy, histology (Marsh criteria), and symptom data collected retrospectively. The AC group included patients newly diagnosed with CeD, or diagnosed within 6 months with ongoing elevated tTG-IgA, and known to have ongoing significant gluten exposure. The CeDGFD group included patients who had been following a gluten-free diet for at least 6 months and based on clinical assessments had no known gluten exposure, along with down-trending tissue transglutaminase IgA. The control group had patients who did not have CeD but had undergone lactulose–mannitol intestinal permeability testing as part of the evaluation for ongoing GI symptoms. Patients were excluded if they had IBD, duodenitis, or if enough samples were not available. The first time point for intestinal permeability testing was used for consistency, as the second time point was not available for all.

### Lactulose–mannitol intestinal permeability testing

The lactulose–mannitol intestinal permeability test was performed as described previously (22, 23). After overnight fasting, subjects ingested a solution containing 5 g lactulose, 2 g mannitol, and 5 g of sucrose in 100 mL water. Urine was collected at two time points in a specimen container. The first urine sample, considered the baseline (0 h), was collected before drinking the oral sugar solution. The second urine sample was collected 2 h after administration of the sugar solution. The urine samples were centrifuged at 3,000 RPM for 5 min, and the concentration of lactulose and mannitol was measured in the supernatant using an enzymatic method. Intestinal permeability was expressed as the lactulose-to-mannitol ratio (LMR = % lactulose excreted/% mannitol excreted).

### Quantification of FABP2 in urine samples

Urine samples were centrifuged at 4°C for 5 min at 13,000 rpm, and the clear supernatant was used to measure FABP2 levels. FABP2 protein concentrations were measured using a commercially available enzyme-linked immunosorbent assay (ELISA) kit (Abcam, Inc., Waltham, MA, USA), following manufacturer's instructions. Briefly, 50 µL of the urine sample or standards and 50 µL of capture and detector antibody cocktail (HRP conjugated) were added to a SimpleStep ELISA® plate (Abcam, Inc Waltham, Massachusetts, USA) and incubated for 60 min on a plate shaker set at 400 rpm. The plate was then washed three times with the 300 µL of wash buffer, and 100 µL of TMB (3,3′, 5,5′ tetramethylbenzidine) development solution was added to each well. It was then incubated for 10 min in the dark on a plate shaker set at 400 rpm. Stop solution of 100 µL was then added and the O.D was read at 450 nm. The concentration of FABP2 was calculated from the standard curve and expressed as pg/mL.

### Statistical analyses

Data are reported as medians with interquartile ranges, and frequency is reported as percentages. Fisher's exact tests were conducted for categorical variables, while Kruskal–Wallis tests followed by Dunn's multiple comparisons were applied for continuous non-parametric analysis. Finally, receiver operating characteristic (ROC) curve was generated utilizing FABP2 data to evaluate its diagnostic testing. Statistical analysis was performed using R Statistical Software v4.3.2 (24) and tables were generated using the Table 1 R package v 1.4.3 (25, 26). GraphPad Prism Software v9.5 was used to prepare the graphs.

## Results

### Patient demographics and clinical characteristics

In this retrospective study, urine samples from 103 pediatric patients who underwent lactulose/mannitol intestinal permeability testing were analyzed. Patients were grouped as AC, CeDGFD, and controls. The AC group was diagnosed using a combination of endoscopy and elevated serology ≥ 10 U/L (2.8 to ≥10 U/L), with one patient diagnosed as per European Society for pediatric gastroenterology hepatology and nutrition (ESPGHAN) criteria, without biopsy (8).

The median duration on a GFD since CeD diagnosis in the CeDGFD group was 2.2 years (range 1.5–5 years).

Of the patients in the CeDGFD group, 13 had tTg-IgA levels greater than their respective cutoffs, though all values had trended downward from baseline. The median time between intestinal permeability testing and serology was 26 days (7–85). The mean (SD) age was 12.0 years (9.72–15.1). Overall, patients with AC were significantly younger than those with CeDGFD and controls (*p* = 0.028). A total of 58.3% (60/103) were girls and 41.7% (43/103) were boys (Table 1).

**Table 1:** Demographic data of patients in AC, CeDGFD, and control groups.

Demographics	Overall	Active celiac disease	Celiac on GFD	Control	*p*-value
	(*N* = 103)	(*N* = 12)	(*N* = 26)	(*N* = 65)	
Sex					
Male	43 (41.7%)	5 (41.7%)	7 (26.9%)	31 (47.7%)	0.205
Female	60 (58.3%)	7 (58.3%)	19 (73.1%)	34 (52.3%)	
Age (years)	12.0 [9.72 to 15.1]	10.1 [6.51 to 10.6]	12.4 [10.7 to 16.0]	12.8 [9.32 to 15.2]	0.028
L/M ratio	0.04 [0.02 to 0.05]	0.06 [0.03 to 0.12]	0.03 [0.03 to 0.04]	0.03 [0.02 to 0.05]	0.039
Height (Z-score)	−0.19 [−0.81 to 0.55]	−0.17 [−0.62 to 0.35]	0.07 [−0.62 to 0.54]	−0.32 [−0.86 to 0.49]	0.817
Weight (Z-score)	0.06 [−0.85 to 0.74]	0.03 [−1.07 to 0.66]	−0.10 [−0.90 to 0.32]	0.12 [−0.67 to 1.09]	0.295
BMI (Z-score)	0.14 [−0.61 to 1.15]	−0.19 [−0.56 to 1.47]	−0.21 [−0.66 to 0.58]	0.27 [−0.45 to 1.31]	0.098

For categorical variables, patient data are presented as absolute numbers with percentages in parenthesis. The overall % was calculated from the total number of cases (*N* = 103) and the percentages of each subgroup were calculated from the overall number of cases in that group. *p-*values were calculated using Fisher's exact tests, with significance defined as *p* < 0.05. Numeric variables are presented as Median [Quartile 1 to Quartile 3]. For numeric variables, *p*-values were calculated using Kruskal–Wallis tests, and Dunn's multiple comparisons tests were performed for *post hoc* analysis.

The clinical characteristics of patients in the AC, CeDGFD, and control groups are summarized in Table 2. The most common clinical symptoms in this cohort were abdominal pain (82/103, 79.6%) and constipation (66/103, 64.1%) (Table 2). Short stature was more commonly seen in the AC group compared with controls (*p* < 0.05). Symptoms such as abdominal pain, constipation, and nausea were seen more commonly present in controls compared with the CeDGFD group (*p* < 0.05) (Table 2).

**Table 2:** Clinical symptoms of patients in AC, CeDGFD, and control groups.

Clinical symptoms	Overall	Active celiac disease	Celiac on GFD	Control	*p*-value
	(*N* = 103)	(*N* = 12)	(*N* = 26)	(*N* = 65)	
Abdominal pain	82 (79.6%)	9 (75.0%)	13 (50.0%)	60 (92.3%)	<0.001
Anxiety	10 (9.7%)	0 (0%)	4 (15.4%)	6 (9.2%)	0.357
Constipation	66 (64.1%)	10 (83.3%)	10 (38.5%)	46 (70.8%)	0.006
Diarrhea	17 (16.5%)	1 (8.3%)	2 (7.7%)	14 (21.5%)	0.266
Faltering growth	8 (7.8%)	1 (8.3%)	1 (3.8%)	6 (9.2%)	0.758
Gas	14 (13.6%)	2 (16.7%)	3 (11.5%)	9 (13.8%)	0.83
Nausea	19 (18.4%)	0 (0%)	1 (3.8%)	18 (27.7%)	0.011
Short	6 (5.8%)	3 (25.0%)	2 (7.7%)	1 (1.5%)	0.008
Weight loss	9 (8.7%)	1 (8.3%)	5 (19.2%)	3 (4.6%)	0.074
Asymptomatic	2 (1.9%)	0 (0%)	1 (3.8%)	1 (1.5%)	0.585

Data for patients in each group are presented in absolute numbers with percentage in parenthesis. The overall % was calculated from the total number of cases (*N* = 103) and the percentages of each subgroup were calculated from the overall number of cases in that group. Many of these clinical characteristics co-existed. *p-*values were calculated using Fisher's exact tests, with significance defined as *p* < 0.05.

### Lactulose/mannitol intestinal permeability (leaky gut) results

The concentration of lactulose and mannitol was measured in all the urine samples collected and was expressed as the lactulose-to-mannitol ratio (L/M). The lactulose/mannitol leaky gut ratio was significantly higher in the AC group (median L/M = 0.06) compared with both the CeDGFD group (median L/M = 0.03) and controls (median L/M = 0.03) (*p* = 0.039) (Table 1).

### Quantification of FABP2 in urine samples of study cohort

**Figure 1:**
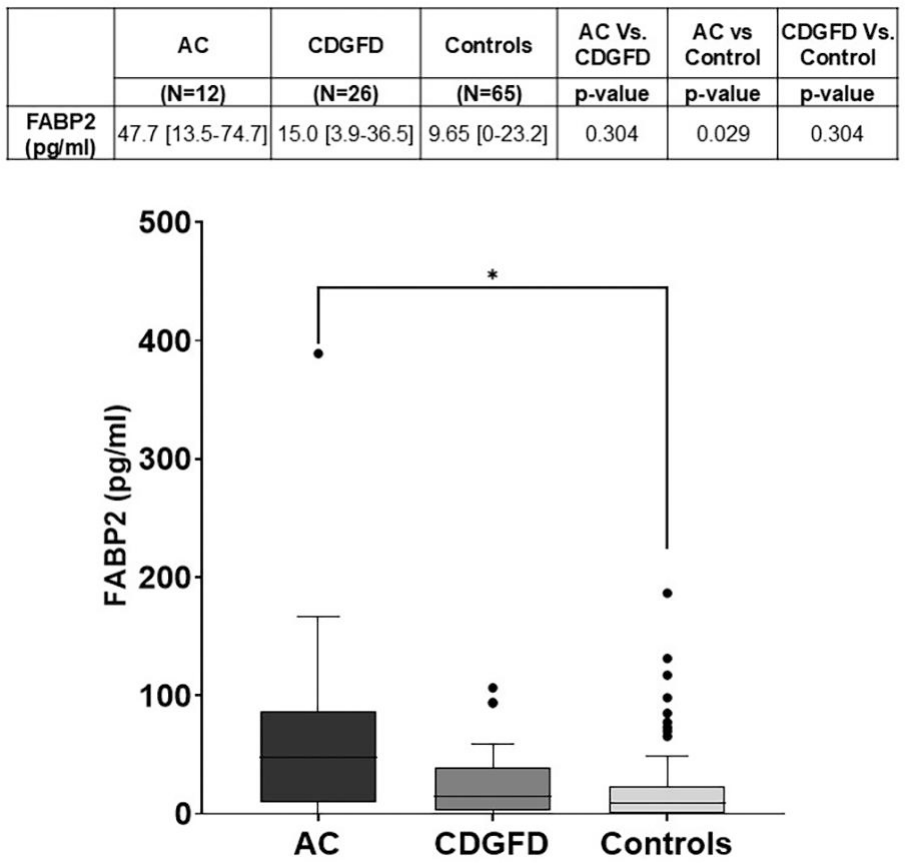
The levels of FABP2 in urine samples of patients with active celiac disease (AC) were higher compared with patients with celiac disease on gluten-free diet (CeDGFD) and controls. Statistical significance was calculated using Kruskal–Wallis tests (*p-*value < 0.05). Dunn's multiple comparisons tests were performed for *post hoc* analysis.

Median FABP2 levels were significantly higher in AC patients than controls [47.7 pg/mL (13.5–74.7) vs. 9.65 pg/mL (0–23.2) (*p* = 0.029)]. In CeDGFD patients, FABP2 levels showed a downward trend, with a median of 15.0 pg/mL compared with 47.7 pg/mL in the AC group; however, this difference was not statistically significant (*p* > 0.05). Similarly, FABP2 levels in the CeDGFD group were not significantly different from those in controls (*p* > 0.05) (Figure 1).

### Receiver operating characteristics curve analysis

**Figure 2:**
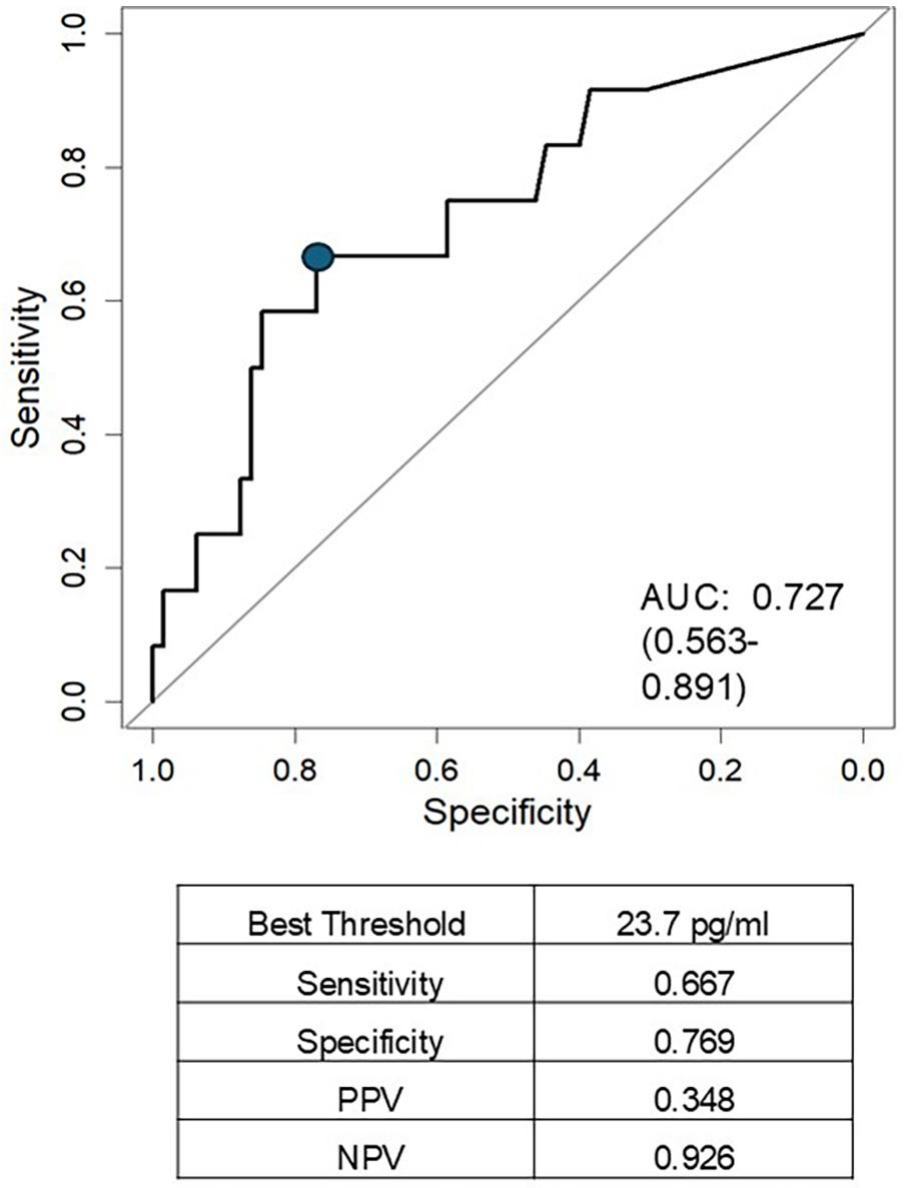
ROC curve analysis shows that the best threshold to differentiate between patients with AC and those without celiac diseases was 23.7 pg/mL, with a sensitivity of 66.7% and a specificity of 76.9%.

To evaluate the diagnostic performance of FABP2 in distinguishing active celiac disease from controls, a ROC curve analysis was performed. The area under the curve was determined to be 0.727 (0.563–0.891). An optimal threshold of 23.7 pg/mL of FABP2 provided a sensitivity of 0.67 and a specificity of 0.77. At this threshold, the positive predictive value (PPV) was 0.348 and the negative predictive value (NPV) was 0.926 (Figure 2).

## Discussion

Our study demonstrates utility of urine intestinal FABP2 as a biomarker for the evaluation and diagnosis of CeD, with potential to serve as a longitudinal marker for mucosal healing. ROC curve analysis confirmed the diagnostic utility of FABP2 as a biomarker for celiac disease, with an area under the curve (AUC) of 0.727 (95% CI = 0.563–0.891). With a cutoff value of 23.7 pg/mL of FABP2, the sensitivity and specificity of this biomarker were determined to be 66.7% and 76.9%, respectively, which helped differentiate patients with active celiac disease from those without celiac disease.

FABP2 is a small cytosolic protein predominantly expressed in mature enterocytes of the small intestine, where it facilitates intracellular fatty acid transport and lipid metabolism (27). In intestinal diseases such as active celiac disease, epithelial injury leads to FABP2 release into circulation. Given its low molecular weight, FABP2 is freely filtered by the kidneys and subsequently appears in urine. Its short circulating half-life allows it to reflect ongoing epithelial damage; as mucosal integrity improves, with adherence to a gluten-free diet, circulating and urinary FABP2 levels decline, supporting its utility as a dynamic, non-invasive biomarker for disease activity and mucosal healing.

At present, tTG-IgA and endomysial antibody (EMA) offer high sensitivity and specificity in the evaluation of celiac disease (28). However, these tests can provide false-negative results in patients with selective IgA deficiency, and they can also be labor-intensive and expensive. Upper GI endoscopy with histological findings suggestive of celiac disease remains the gold standard for diagnosis. Individuals with positive serology but minimal or no histological changes (Marsh 0 or Marsh 1) may be classified as having potential celiac disease; however, this approach is invasive and requires sedation.

Therefore, an additional non-invasive marker that is cost-effective and provides improved patient comfort would be desirable. Thus, the inclusion of FABP2 in celiac disease screening may help identify intestinal injury and may lead to better diagnostic outcomes before proceeding to endoscopy.

Follow-up in celiac disease typically requires serial monitoring of tissue transglutaminase IgA until normalization. The time frame for normalization is variable, with one study reporting that the majority of patients adhering to a gluten-free diet required more than 1 year for normalization (29). Furthermore, a meta-analysis evaluating these tests in pediatric and adult populations with CeD found that serologic markers correlate poorly with mucosal healing following adherence to a strict gluten-free diet (30).

Our findings align with established serum FABP2 studies. Kaul et al. evaluated diagnostic performance in 98 Indian children (74 with confirmed CeD), reporting 51% sensitivity and 88% specificity at a 920 pg/mL cutoff (31). Derikx et al. demonstrated in a pilot study that circulating FABP2 levels are significantly elevated in untreated CeD and normalize following adherence to a gluten-free diet (32).

There is a need for tests that reflect short-term variations, whether indicating recovery or gluten exposure, to effectively monitor intestinal mucosal normalization and adherence to a gluten-free diet. In one study assessing serum FABP2 in adults, five patients with celiac disease who underwent repeat endoscopy at an average of 19 months after initiating a gluten-free diet exhibited persistent intestinal damage reflected by elevated FABP2 levels, despite normalization of tTG -IgA levels in two cases (14). Adriaanse et al. demonstrated that serum FABP2 increases significantly during a 2-week gluten challenge, whereas autoantibody levels show most pronounced elevation at day 28 (33). In our study, FABP2 levels were lower in the CeDGFD group but did not differ significantly from either AC or controls, likely due to the small sample size, age, or continued intestinal damage in some patients, despite being on a GFD (AC younger, *p* = 0.028). Lactulose/mannitol intestinal permeability testing had normalized in both control and CeDGFD groups. The median time at follow-up for the CeDGFD group was 2.2 years. We used the intestinal permeability test as a marker of normalized histology on a gluten-free diet, as we did not have repeat histology to confirm the findings. Previous studies have demonstrated intestinal permeability testing to be a sensitive marker for healing in CeD on a gluten-free diet (7, 34–36). However, as lactulose/mannitol intestinal permeability testing is time-consuming and time-sensitive, typically conducted 2–3 h post-dose, future prospective studies with FABP2 could establish both markers’ roles in assessing mucosal health and offer better tracking over time.

This study's limitations include the use of historical samples that had been frozen, which may have degraded over time, and limited sample size, constraining the statistical power and interpretability of our findings. In addition, we did not have urine creatine levels to normalize FABP2 concentrations. However, a previous study found no significant difference when normalizing urine FABP2 for creatinine (19). Moreover, tTG-IgA measurements were not collected in close temporal proximity to intestinal permeability testing. While both tTG-IgA and FABP2 may trend downward with gluten-free diet adherence, their normalization timelines likely differ. For example, a previous study reported that 92% of individuals normalized serum FABP2 within 26 weeks (37). The extent to which urine FABP2 correlates with mucosal healing remains uncertain as biopsy data were not uniformly available at time points corresponding to urine sampling, due to the retrospective nature of the study. Establishing such correlations would have required repeat endoscopy with histological evaluation, which was not feasible in this cohort. Utilizing appropriate clinical controls with abdominal discomfort provides a reasonable comparison. However, because the control individuals underwent intestinal permeability testing for abdominal discomfort, their FABP2 levels may have been higher than would otherwise be expected.

Future prospective studies should include a healthy control group comprising individuals without intestinal disease or symptoms. Serial measurements of serum and urine FABP2 would allow correlation with mucosal status. Although repeated histological assessment would provide the most definitive validation, practical constraints may necessitate continued reliance on urinary intestinal permeability assays for comparison. Extending this research to adult populations where repeat endoscopy and biopsy are more feasible would further strengthen the evidence base.

In conclusion, our study shows intestinal fatty acid binding protein increases with intestinal damage in celiac disease at diagnosis, with a potential role to follow normalization of histology track response and compliance of gluten-free diet. This biomarker has the potential to serve as a minimally invasive tool for detecting persistent mucosal abnormalities, reducing the need for repeated endoscopic evaluations. These findings lay the foundation for future prospective studies to further validate this biomarker for celiac disease diagnosis and monitoring.

## Data Availability

The original contributions presented in the study are included in the article/Supplementary Material; further inquiries can be directed to the corresponding author.
